# The wonder exerkines—novel insights: a critical state-of-the-art review

**DOI:** 10.1007/s11010-021-04264-5

**Published:** 2021-09-23

**Authors:** Laura Magliulo, Danilo Bondi, Niccolò Pini, Lorenzo Marramiero, Ester Sara Di Filippo

**Affiliations:** Department of Neuroscience, Imaging and Clinical Sciences, University “G. d’Annunzio” of Chieti - Pescara, Chieti, Italy

**Keywords:** Physical exercise, Organokines, Exosomes, miRNA, Cross-talk, Myokines

## Abstract

Several benefits can be acquired through physical exercise. Different classes of biomolecules are responsible for the cross-talk between distant organs. The secretome of skeletal muscles, and more widely the field of organokines, is ever-expanding. “Exerkine” has emerged as the umbrella term covering any humoral factors secreted into circulation by tissues in response to exercise. This review aims at describing the most interesting exerkines discovered in the last 3 years, which are paving the way for both physiological novel insights and potential medical strategies. The five exerkines identified all play a significant role in the healthy effect of exercise. Specifically: miR-1192, released by muscles and myocardium into circulation, by modulating cardioprotective effect in trained mice; miR-342-5p, located into exosomes from vascular endothelial cells, also a cardioprotective miRNA in trained young humans; apelin, released by muscles into circulation, involved in anti-inflammatory pathways and muscle regenerative capacity in rats; GDF-15, released into circulation from yet unknown source, whose effects can be observed on multiple organs in young men after a single bout of exercise; oxytocin, released by myoblasts and myotubes, with autocrine and paracrine functions in myotubes. The systemic transport by vesicles and the crosstalk between distant organs deserve a deep investigation. Sources, targets, transport mechanisms, biological roles, population samples, frequency, intensity, time and type of exercise should be considered for the characterization of existing and novel exerkines. The “exercise is medicine” framework should include exerkines in favor of novel insights for public health.

## Background

Physical exercise is associated with a large number of beneficial effects. Exercise-related adaptations include, among the others, cardiovascular, nervous, metabolic, locomotor, immune, and respiratory system [1]. Physical exercise represents a powerful tool, doable with few or no side effects and produces a multitude of benefits at the same time.

### Exercise and exerkines

But how does the exercise adaptation work? During exercise there is a crosstalk among many organs and cells, mediated by many biomolecules secreted in response to exercise. In the early 2000s, the concept of myokine was introduced to describe cytokines released by muscle to exert autocrine, paracrine and endocrine effects [2, 3]. Since then, the role of skeletal muscle as the largest secretory organ has been defined, with a growing body of evidence on the muscle-organ cross-talk and with the identification of specific myokines [3]. The secretome of exercising skeletal muscle has the power to act through endocrine signaling mediators, spreading specific effects on muscular tissue itself and on epithelial, connective and nervous tissues. As Hoffman and Weigert [4] pointed out, both myofibers and satellite cells, fibroblasts, immune cells, endothelial cells, and extracellular matrix contribute to the muscle secretome, whose effects, in addition to muscle themselves, range from inflammatory, and immune system, to bones and brain. Even better, exercise-related muscle factors may have effects on almost all cell types and organs [4]. However, several biomolecules are released from non-muscle tissues as acute or chronic response to physical exercise. Therefore, the term “exerkines” now is used to describe those humoral factors (peptides, metabolites and RNAs) secreted into circulation by any organ in response to acute exercise or exercise training [5]. Exerkines can be directly secreted into circulation or can be transported by extracellular vehicles (EVs) such as exosomes. Molecular targets and receptors for exerkines are found throughout the body, including skeletal muscle, fat, liver, pancreas, bone, heart, immune, and brain cells.

The first exerkine that was by lucky chance discovered, belonging to the class of myokines, was interleukin 6 (IL-6), which rapidly increases in the blood after physical activity. Later, other myokines such as musclin, IL-15, apelin,a secreted protein acidic and rich in cysteine, myonectin, fibroblast growth factor 21 (FGF-21), decorin and irisin have been discovered to be differently modulated in circulation in response to exercise. Some of these (i.e., IL-6, IL-7, myostatin) exert their effects directly on muscle tissues and are involved in the control of muscle mass, proliferation and muscle repair. Others, such as irisin and myonectin, appear to have systemic effects by playing a critical role in the modulation of the metabolic system, exercising their effect on liver, the adipose system, pancreas, and in the modulation of the immune system. On the contrary, the osteogenic factors insulin-like growth factor-1 (IGF-1), FGF-2 and Follistatin-related protein 1 released after exercise, improve endothelial function of vascular system, with a significant relevance of physical activity in cardiovascular disease. Recent studies are looking at the possible pharmacological use of myokines targets, as well as physical activity, to counteract the progression and consequences of diseases such as cancer cachexia [6], diabetes, heart failure and chronic obstructive pulmonary disease [7].

Besides the myokines, over the last 30 years, there has been much evidence on a “relative-new class” of genes called micro RNAs (miRNAs). miRNAs are non-codifying small RNAs that have emerged as powerful agents that control the expression of gene pool and lead to post-transcriptional regulation [8]. These miRNAs are released from several types of cells across the body, directly on the body fluids stream or vesiculated through nano-vesicles, acting as important intercellular communicators. Several studies have reported the association of changes in circulating miRNAs levels in many diseases (appearing as promising clinical biomarkers) but also as consequences of physiological adaptation to a stimulus, for example during exercise.

In fact, miRNAs have been found to be differently expressed after physical activity both in humans and animals. A large amount of miRNAs modulate the exercise-related cardiovascular adaptations [9]. Others regulate muscular hypertrophy and regeneration (e.g., miR-1, miR-27a/b, miR-29, miR-146a, miR-133 miR-206, miR-675–3/5p) [10]. Circulating miRNAs are involved in “anabolic resistance” [11], angiogenesis, neuronal regeneration and metabolism [9]. The observations of miRNAs as exercise-related factors have important implications for the understanding of how to maintain health throughout the lifespan.

Many of the biomolecules differentially modulated during exercise are included in the “organokines” class. Organokines, as messenger peptides interacting with each other, provide crosstalk between tissues via autocrine, paracrine or endocrine action [12]. This framework deals with the network among exercise, gut and immune system, with the ever-expanding universe of gut microbiota [13]. In addition to muscle, two other sources of organokines have been extensively studied: adipose system and liver. Saeidi et al. [14] recently pointed out the role of adipokines in mediating the beneficial effect of physical activity facing with overweight and obesity. They reviewed several adipokines, highlighting the role of TNF-α, IL-6, adiponectin, visfatin, omentin-1 and leptin. Ennequin et al. [15] largely reviewed the exercise-induced liver secretome, observing that it consists primarily in IL-6, FGF-21, Fetuin-A, ANGPTL4, and Fst. They argue that these hepatokines may act as a conduit for acute and chronic adaptation to exercise and may participate in inter-organ crosstalk. The plethora of exercise-induced hepatokines could be extended to HSP-72, IGF-1 and IGFBP1, as pointed out by the review of Weigert et al. [16].

### Cross-talk across systems and organs

**Fig. 1:**
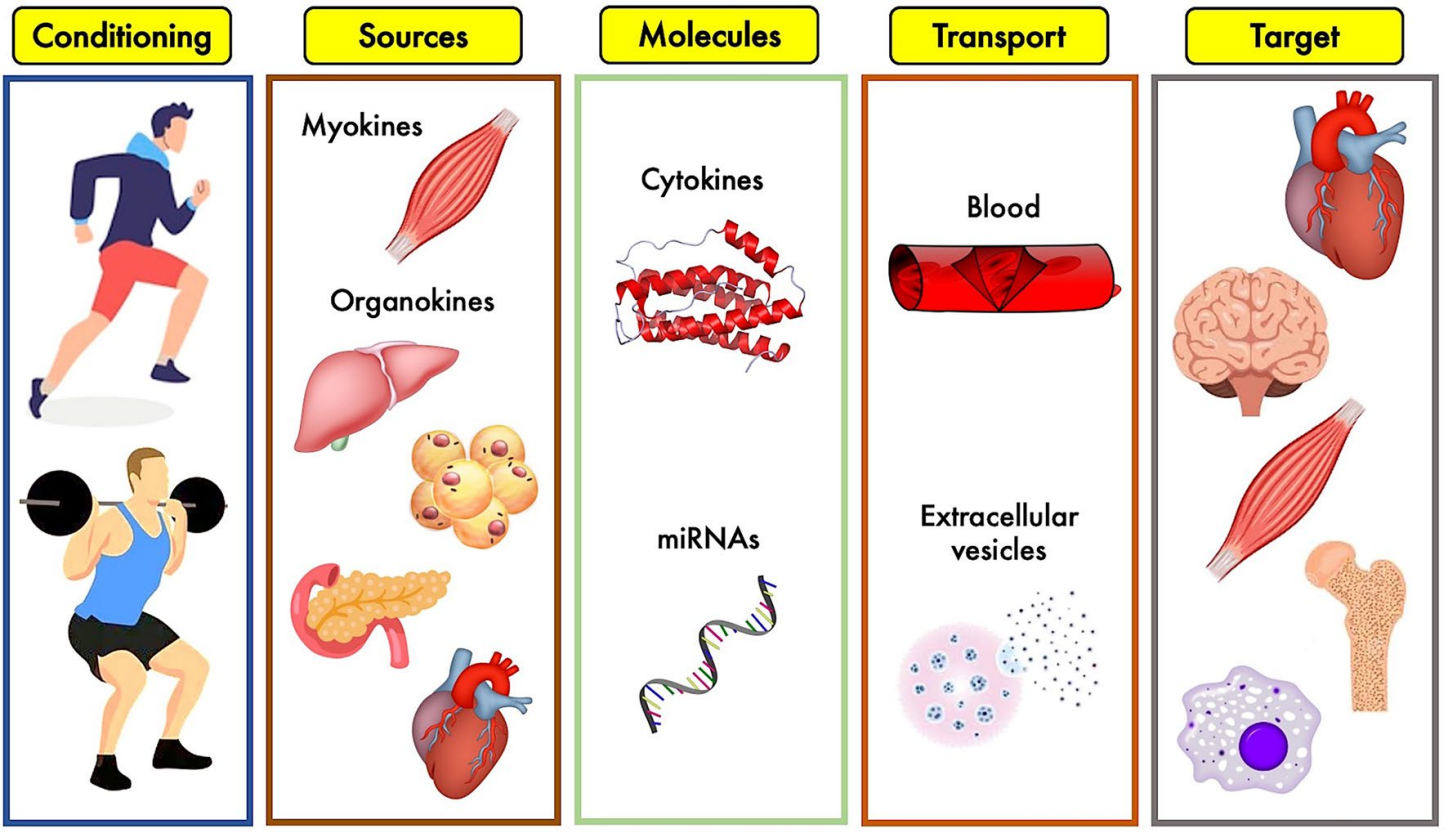
Overview of the exerkines’ system: different types of physical exercise stimulate biological tissues to release a plethora of cytokines and other molecules which, circulating into blood stream or stuffed into extracellular vesicles, reach biological targets to produce specific effects

Understanding the role of exerkines does not mean to just focus on the effects of the single molecule on a specific pathway but also on the analysis of the effects of the same system over organs (Fig. 1). In this complicate ever-expanding universe, over the years a special focus has been posed on the effects of exercise to nervous system; in particular, the correlation between post-exertional fatigue and cytokines has been studied. The central nervous system is extremely sensitive to specific cytokines like IL-1, IL-6, and TNF [17]. The brain-derived neurotrophic factor (BDNF) has been the most extensively studied factor; e.g., Liu and Nusslock [18] reviewed the role of BDNF in exercise-related neurogenesis. Several other exerkines can affect brain health, such as the myokines irisin and cathepsin B, the bone-derived hormone osteocalcin, the adipokines leptin and adiponectin, the hepatokines FGF-21 and IGF-1 [19]. Among these, IGF-1 has been extensively studied as neurotrophic factor, in response to aerobic and resistance exercises, explicating its function not only on the central nervous system but on the peripheral system as well [20].

Provided these considerations, one of the main questions regarding the transport mechanism still stands. Many studies analyzed how these molecules can affect each other and interact with systems and sub-systems, focusing on the exerkines’ transport. It has been hypothesized that many exerkines may be contained into extracellular vesicles (EVs) to exert their endocrine-like communication [21]. EVs are small endogenous membrane vesicles secreted by most cell types. It has been observed that they play an important role in mediating cell-to-cell communication and crosstalk between organs via the transmission of a variety of signaling molecules including proteins, mRNAs, cytokines, metabolites, many of which are differently modulated during exercise, as previously described. Although the biogenesis and transport of exercise-related EVs is still unclear, the intracellular increase of calcium is associated with the release from cells [22]. Therefore, a rapid increase in EVs trafficking during exercise has been suggested to be an important pathways for inter-tissue cross-talk [23]. Over the last 30 years, the investigation has tried to explain the role of extracellular vesicle in exercise-mediated cross-talk with a particular focus on skeletal muscle: it was demonstrated that EVs release is mediated by calcium concentration in cells. During the exercise, due the massive release of calcium from the sarcoplasmic reticulum, after motoneuronal stimulation on skeletal muscle fibers, most of the EVs are released from muscle cells [24]. On the other hand, the EVs cargo change in relation of the physical activities, also depending on the kind of exercise (i.e., aerobic vs anaerobic) [25]. Furthermore, the uniqueness of a pool of exosomal miRNAs following acute exercise has been demonstrated [26]. Whitham and colleagues [22] found an increase of more than 300 proteins in the circulation, many of them released by EVs, identifying 35 new possible myokines, also found in myotubes cell conditioned medium.

The possibility of investigating the topic of exerkines in human, rather than animal models, assumes a crucial role in the theoretical understanding and applicative perspectives. As for the population sample, most studies dealt with elderly. As a matter of fact, aging is marked by a plethora of biological pathways, such as sarcopenia, i.e., the loss of muscle mass and function with age. Within this framework, physical activity represents a key point in the multifaceted approach to deal with this public issue, which crucially impairs the quality of life of elderly [27]. Barbalho et al. [28] recently pointed out the role of myokines as protagonists in mediating the beneficial effects of physical exercise against sarcopenia. The molecular and neural plasticity in response to physical exercise in elderly rises the role of exerkines in mediating the exercise-related neuroprotection, especially in case of aerobic exercise [29].

### Aim of this review

Given these premises, the present review points out the most recent and promising advancements in the topic of organ-derived exercise factors, acting on the organ itself or cross-talking with other organs. We designed a critical and concise review on a chronological basis, starting from the historical context and reporting the most promising evidence arbitrarily since 2016 in the following sections. The authors aimed to review the novel advancements into the field of exerkines, summarizing those molecules which have the potential to be included along with the most robust evidence of exerkines, providing fascinating insights for future research in next years.

## Recent advancements in exerkines' world

**Fig. 2:**
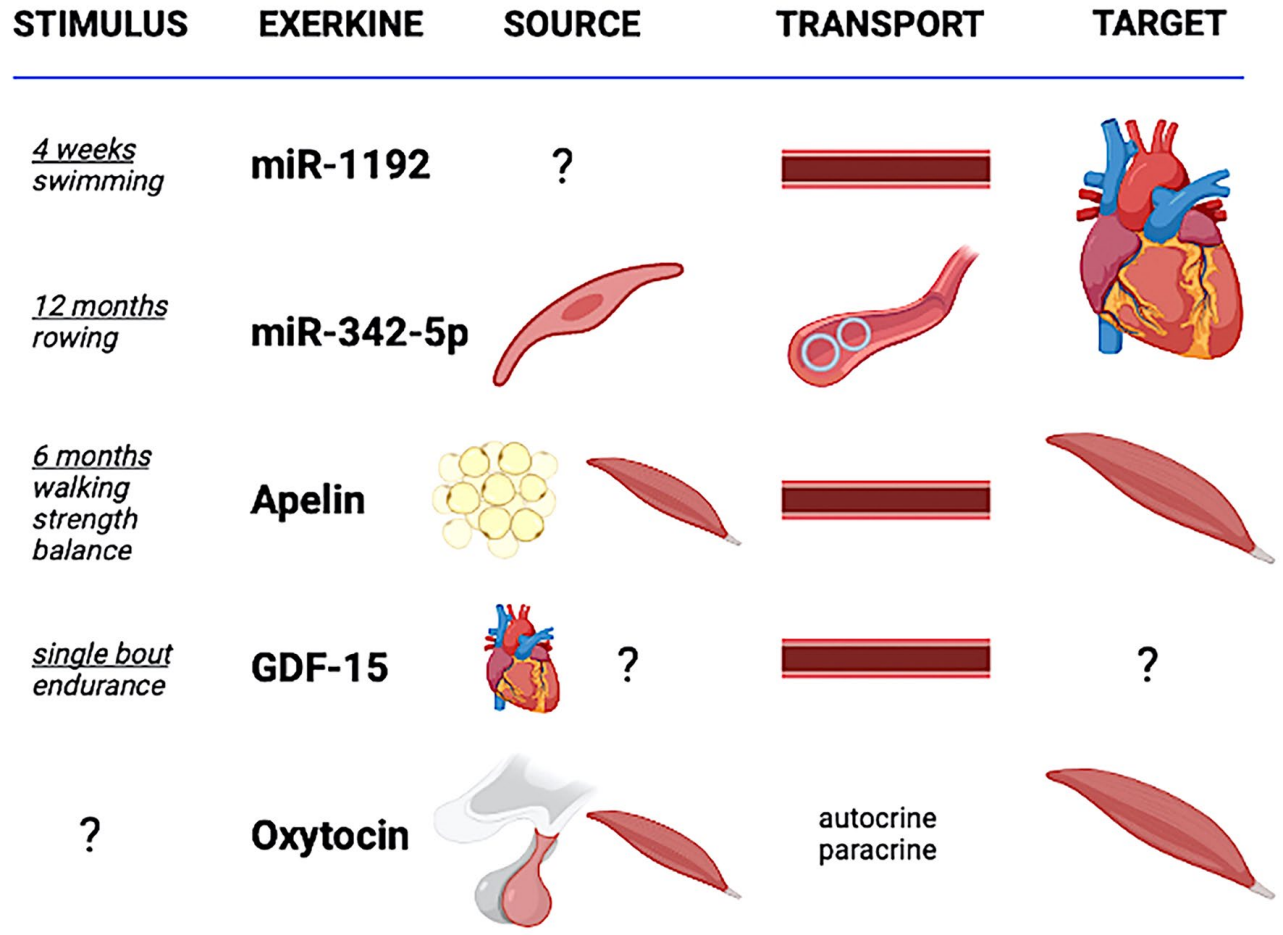
Graphical description of the five novel exerkines' framework. The source of release of miR-1192 is still under debate; miR-342-5p is released by vascular endothelial cells and stuffed into circulating vesicles; apelin is released by adipose and muscle cells; oxytocin is released by posterior pituitary gland and muscle cells, and likely acts through autocrine and paracrine processes; GDF-15 is released from heart and likely from other sources, but not from muscles. Both miR-1192, apelin and GDF-15 are transported by blood stream. The main target of both miR-1192 and miR-342-5p is heart, the one of both apelin and oxytocin is muscle, while the target of GDF-15 is still unclear

It has been extensively demonstrated that exercise training is a valuable strategy to stay healthy and counteract many diseases. However, the mechanisms underlying the protective effects of exercise are still unclear. One of the mechanisms that have been proposed to be responsible for exercise-induced protection, including the different expression of this training-related mediators, is based on exerkines. Despite the difficulty in revealing the complex mechanism hidden behind these events, in the last 3 years, different studies have brought to light new molecules classifiable under the term of exerkines (Table 1, Fig. 2). We will describe the main findings for what concerns “novel exerkines” below.

**Table 1:** Recent advancements in the findings about exerkines

Study	Exerkine	Condition	Source	Transport	Effect	Target
Wang et al. [30]	miR-1192	4-week of swim training, in mices	Muscles and myocardium?	Circulation	Cardioprotective	Caspase3
Hou et al. [33]	miR-342-5p	Rowing training for over 1-year, in 19–22-years old students	Vascular endothelial cells	Exosomes	Cardioprotective	Capsase9 and Jnk2
Vinel et al. [44]	Apelin	Daily 30-min bout of endurance exercise, 6 days/week, 28 days, in rats	Muscles	Intracellular, circulation	Mitochondriogenesis, autophagy, and anti-inflammatory pathways in myofibers, muscle regenerative capacity	APJ receptor
Kleinert et al. [47]	GDF-15	60-min endurance exercise bout, in 27-years old men	Not the muscles	Circulation	Multiple organ stimulation	GFRAL receptor
Berio et al. [54]	Oxytocin	Muscle cell line C2C12	Myoblasts and myotubes	Autocrine and paracrine?	Hormonal regulation, skeletal muscle metabolism?	OXT receptor

*miR* micro-RNA, *GDF* growth differentiation factor, *Jnk2* c-Jun N-terminal kinase 2, *APJ* orphan G protein–coupled apelin receptor, *GFRAL* glial-derived neurotrophic factor receptor α-like, *OXT* oxytocin

### miR-1192

Starting with the most recent, we begin describing Wang et al.s’ work [30]. They found out that 4-week swimming training exerted a protective effect against myocardial infarction in mice. From analysis of plasma, they found ten differentially expressed miRNAs. Among them, miR-1192 was increased after exercise and it exerted significant protective effect against hypoxia in cultured neonatal cardiomyocyte via targeting Caspase 3. In order to evaluate the effective protective capacity of miR-1192, in the same work, they evaluated the effect of injection of agomiR-1192, exerting similar cardioprotective action, while inhibition of that miRNA abolished the cardioprotective effect of exercise in myocardial infarction. Riding the wave of previous studies, that have shown the exercise-related cardioprotective effect of other miRNAs, such as miR-1, miR-17-3p, miR-29a, miR-29c, miR-214, and miR-222 [31], miR-1192 can be considered a novel exerkine to be taken into account for cardiac protection. Besides heart, miR-1192 is expressed in muscle tissue, where it inhibits the myogenic potential, with a mechanism suppressed by the Human antigen R (HuR) to helps undifferentiated muscle cells to enter myogenesis [32]. Therefore, the possible beneficial effect of miR-1192 should be considered only targeting heart tissue.

### miR-342-5p

In 2019, Hou et al. [33] investigated the cardioprotective effects of long-term exercise (team-based rowing training for over 1 year) against myocardial ischemia/reperfusion injury. Particularly, they discovered that physical activity increased the release of exosomes in plasma both in animals and humans. Analyzing the exosomes cargo, they found a differently expressed 12 miRNAs, among which miR-342-5p was not only significantly increased but also exerting profound cardioprotective effects both in vitro and in vivo. However, the role of miR-342-5p seems controversial. On the detrimental side of the coin, serum miR-342-5p has been suggested as a biomarker for adverse cardiovascular events [34, 35]. Indeed, miR-342-5p may promote cell proliferation, migration and invasion of vascular smooth muscle cells, via the Akt signaling pathway [36]. On the bright side, miR-342-5p modulates the cardioprotective effect by inhibiting cardiomyocyte apoptotic signal mediated by Caspase 9 and c-Jun N-terminal kinase 2 (Jnk2) and enhances survival signaling (p-Akt) in the ischemic heart [33]. Therefore, the role of miR-342-5p to the cardiovascular system may be conversely exerted depending on the transport system, whether `ly circulating or stuffed into vesicles.

### Apelin

Apelin is a peptide of 13–36 amino acids that was identified in 1998 [37]: it is an endogenous ligand for apelin receptor (orphan G protein–coupled APJ receptor), widely expressed in various organs such as skeletal muscle, heart, lung, kidney, liver, adipose system, gastrointestinal tract, brain, adrenal glands, endothelium, and human plasma. Depending on the target, apelin can exert distinct functions, such as control of blood pressure, stimulation of cardiac contractility, regulation of water and food intake, adipocyte differentiation, and bone formation. In skeletal muscles, the apelin receptor present on muscle stem cells promotes in vitro and in vivo proliferation and differentiation taking part in muscle regeneration. Apelin targets muscle cells during aging, both in human and rodents: it acts on muscle metabolism by activating an AMPK-dependent mitochondria biogenesis, it promotes autophagy and decreases inflammation. Muscle apelin expression decreases with age, suggesting that it may play a role in sarcopenia. However, the physiological role of apelinergic system can be shifted to pathological processes under altered microenvironmental conditions [38]. The important role of apelin in energy metabolism, as an additional player to other adipokines, and the changes related to metabolic diseases [39], as well as to cardiovascular pathophysiology [40] has been pointed out. Moreover, apelin has been shown to be expressed in cancer tissue [41]. As an adipokine, apelin could promote the vascular network development in adipocytes niche, upregulated by hypoxia [38]. Initially studied only as an adipokine [42], apelin has been shown to be released by other tissues, such as muscle. In 2012 it was positively linked to self-reported physical activity in diabetic patients, independently by age, sex and BMI [43]. However, in 2018 Vinel and colleagues identified for the first time the relation between apelin and physical exercise (specifically, moderate-intensity protocols) [44]. In vivo, apelin production by myofibers is stimulated by exercise-associated muscle contraction and for this reason could be used a biomarker for the definition of define successful exercise’s strategies in the elderly to reduce aging. Inflammation can suppress the beneficial pathways associated with muscle apelin and its receptor [44]. Recently, it has been demonstrated that apelin drives the fetal brown adipose system and offspring metabolic health in mice, in response to maternal exercise [45]. Therefore, apelin may represent an additional player in the cross-talk between skeletal muscle and brown-beige adipocytes, along with other factors such as irisin [46]. The use of agonists and antagonists has been therefore discussed, mainly about metabolic diseases [39] and cancer [38]. Apelin, and apelin peptides, have also been suggested as interesting biomarkers of cardiovascular pathologies [38].

### GDF-15

Also in 2018, Kleinert et al. provided the evidence to support the growth differentiation factor 15 (GDF-15) as an exerkine [47]. They tested young healthy males with a single bout of endurance exercise, reporting an increase in plasma level of GDF-15 during exercise and in the recovery phase. They also demonstrated that skeletal muscle was not the source of production. In this vein, evidence exists for GDF-15 as a cardiokine [48]. As a matter of fact, GDFs act through the GDNF family receptor alpha-like (GFRAL) receptor [49]. In 2020, Conte and colleagues [50] found a positive correlation between plasma level of GDF-15 with age, and an inverse correlation with active lifestyle. These authors demonstrated a remarkable increase in plasma GDF-15 after a strenuous bout of endurance exercise. Considering that GDF-15 is responsive to mitochondrial stress, the authors argued for the role of this exerkine as a marker of injury, e.g., to kidney. All in all, GDF-15 is a stress-responsive cytokine, with an articulate pattern of beneficial and harmful functions [48]. The causal association of GDF-15 with some cardiovascular diseases (e.g., coronary artery disease and myocardial infarction), whereas no relationship with others (e.g., ischemic stroke and heart failure), has recently raised the promising role of GDF-15 as a potential biomarker or therapeutic target [51]. Evidence of GDF-15 as a beneficial mediator of metabolic improvement after a 12-weeks aerobic exercise protocol in older adults may also give account of its role as a possible therapeutic target [52]. Evidence exists about GDF-15 in mediating the adipose tissue lipolysis triggered by skeletal muscle contraction [53], raising the role of this exerkine into bioenergetics.

### Oxytocin

In 2017, starting from evidence about the presence of oxytocin receptors in skeletal muscles and basing their assumptions on their findings, Berio and colleagues suggested oxytocin may work in an autocrine and paracrine way to regulate muscle metabolism [54]. This evidence is added to the exercised-induced increase in circulating vasopressin and supports the role of skeletal muscle in secreting neurohypophyseal hormones [55]. Oxytocin has been pointed out as a cardioprotective molecule, acting by reducing inflammation, promoting angiogenesis, and improving metabolic function of cardiomyocytes [56]. Alizadeh and colleagues demonstrated that oxytocin acts as a mediator of anti‐tumor effects of interval exercise training in a mouse model of breast cancer [57]. In this regard, altered expression of oxytocin and its receptor have been specifically linked to various cancers, leading to a likely role of oxytocin system in the neural regulation of carcinogenesis, and therefore opening the way for oxytocin as a possible novel biomarker and/or key for developing preventative and therapeutic strategies [58]. Oxytocin is also an age-specific factor important for muscle tissue regeneration and homeostasis, and may therefore be studied as a therapeutic target to postpone the onset of sarcopenia [59].

## Future directions

Intriguing perspectives based on this new world of insights offered by exerkines are becoming of interest to scholars, pharmacologists, and practitioners. In particular, the “exercise of medicine” paradigm should deeply consider the exerkines’ signaling. The expanding myokinome and, more widely, the organokinome urge the definition of theoretical and applicative models. Within the plethora of interesting topics, the systemic transport by vesicles and the crosstalk between distant organs, even the most difficult to target, as brain, assume a key role. Novel physiological systems are occurring in the world of exerkines.

For example, a topic of interest is the purinergic signaling. Mancinelli and colleagues advocated for a major focus on guanosine-based purines acting on excitable tissues [60]. On this vein, Pietrangelo et al. [61] recently pointed out the role of guanosine-based molecules in the muscle regeneration system. To be specific, free GTP may be released after a muscle damage to stimulate the proliferative boost of muscle stem cells and consequently stimulate the release of exosomes stuffed with guanosine-based molecules. These molecules may be successively transferred to distant organs to exert-specific effects [61]. The chance of purines to enter the brain through the blood–brain barrier, linked to the requirements of purine supply for neuroprotection [62], triggers novel perspectives in the field of muscle-brain cross-talk.

In the world of exerkines, the focus is still primarily on myokines, hepatokines [3], and adipokines [14], but novel insights may come from the investigation of alternative sources of production. Dealing with the world of organokines, many factors interplay in order to regulate homeostasis and pathophysiological pathways (for an overview, see [12]). As a result, a comprehensive understanding of organ crosstalk exercise related will shed a new light for the definition of the mechanistic insights and for the formulation of specific preventive or therapeutic plans. Indeed, organokines and cargo systems are pivotal players into the scientific field of “network physiology” [63], as they account for the interconnection of physiological systems and sub-systems.

For these purposes, we highlight the necessity to describe the sources, targets, transport mechanisms, biological roles, and population samples. The type of exercise should always be defined. There is evidence to suggest that endurance behaves as a more powerful stimulus for the release of exerkines, at least to promote metabolic health and neuroprotection [21, 29]. It has also been proposed that exerkines could be the mechanism behind the cross-transfer of motor functions in elderly [64]. The interplay of several exerkines in mediating the acute and chronic effects of physical activity/exercise, and the diverse factors affecting the pathways of action need to be addressed extensively. Big dataset and large research consortia, such as the promising MoTrPAC [65], are needed in order to provide original and robust insights. Historical and novel exerkines may emerge as interesting biomarkers, preventative or therapeutic agents, to be early implemented; considering the complexity and dynamicity of the mechanisms involved, a patient-oriented evidence that matters (POEM) [66] would be strongly adopted to deal with this possible implementation.

As a matter of fact, the oxidative, inflammatory and neurotrophic mediators vary as a function of exercise type [67]. Thus, defining the FITT (frequency, intensity, time and type) paradigm of exercise training is needed to achieve consensus about exerkine regulation [14]. Practitioners will therefore be able to “sew” specific “dresses” for individuals in exerkine-based manner. Exerkines and exosomes may provide novel insights about mechanisms behind the pathophysiological and physiological pathways related to the exercise-related benefits, and should be taken into account to define new evidence-based “weapons” in favor of public health.

## Data Availability

No datasets were generated or analyzed during the current study.

## Abbreviations

AMPK: Adenosine monophosphate-activated protein kinase
ANGPTLs: Angiopoietin-like proteins
BDNF: Brain derived neurotrophic factor
EV: Extracellular vesicles
FGF: Fibroblast growth factor
FSTL: Follistatin-like protein
GDF: Growth differentiation factor
GTP: Guanosine triphosphate
HSP: Heat shock protein
IGF: Insulin-like growth factor
IL: Interleukin
miRNA: Micro-ribonucleic acid
TNF: Tumor necrosis factor
